# Association of the Somatostatin Analog Octreotide With Magnetic Nanoparticles for Intraocular Delivery: A Possible Approach for the Treatment of Diabetic Retinopathy

**DOI:** 10.3389/fbioe.2020.00144

**Published:** 2020-02-25

**Authors:** Rosario Amato, Martina Giannaccini, Massimo Dal Monte, Maurizio Cammalleri, Alessandro Pini, Vittoria Raffa, Matteo Lulli, Giovanni Casini

**Affiliations:** ^1^Department of Biology, University of Pisa, Pisa, Italy; ^2^Interdepartmental Research Center Nutrafood “Nutraceuticals and Food for Health”, University of Pisa, Pisa, Italy; ^3^Department of Experimental and Clinical Medicine, University of Florence, Florence, Italy; ^4^Department of Experimental and Clinical Biomedical Sciences “Mario Serio”, University of Florence, Florence, Italy

**Keywords:** mammalian retina, pigment epithelium, endothelial cells, retinal explants, apoptosis, biocompatibility, bioactivity

## Abstract

The somatostatin analog octreotide (OCT) displays important neuroprotective and anti-angiogenic properties that could make it an interesting candidate to treat diabetic retinopathy (DR). Unfortunately, systemic drug administration is hindered by severe side effects, therefore topical administration routes are preferable. However, drug delivery through eye drops may be difficult due to ocular barriers and, in the long term, could induce ocular damage. On the other hand, intraocular injections must be repeated to maintain drug concentration, and this may cause severe damage to the eye. To decrease injection frequency, long-term release and reduced biodegradation could be obtained by binding the drug to biodegradable polymeric nanoparticles. In the present study, we made a preparation of OCT bound to magnetic nanoparticles (MNP-OCT) and tested its possible use as an OCT delivery system to treat retinal pathologies such as DR. In particular, *in vitro*, *ex vivo*, and *in vivo* experimental models of the mammalian retina were used to investigate the possible toxicity of MNPs, possible effects of the binding to MNPs on OCT bioactivity, and the localization of MNP-OCT in the retina after intraocular injection. The results showed that, both in human retinal endothelial cells (HRECs) and in mouse retinal explants, MNPs were not toxic and the binding with MNPs did not influence OCT antiangiogenic or antiapoptotic activity. Rather, effects of MNP-OCT were observed at concentrations up to 100-fold (in HRECs) or 10-fold (in mouse retinal explants) lower compared to OCT, indicating that OCT bioactivity was enhanced in MNP-OCT. MNP-OCT in mouse retinas *in vivo* after intraocular delivery were initially localized mainly to the outer retina, at the level of the retinal pigment epithelium, while after 5 days they were observed throughout the retinal thickness. These observations demonstrate that MNP-OCT may be used as an OCT intraocular delivery system that may ensure OCT localization to the retina and enhanced OCT bioactivity. Further studies will be necessary to determine the OCT release rate in the retina and the persistence of drug effects in the long period.

## Introduction

Diabetic retinopathy (DR) is a complication of diabetes that represents one of the major causes of vision loss in humans. The endogenous neuropeptide somatostatin has been widely studied for its powerful neuroprotective properties that may be exploited for DR treatment, and these studies have been extensively reviewed (Gabriel, 2013; Hernandez et al., 2014; Szabadfi et al., 2014; Simo-Servat et al., 2018; Cervia et al., 2019). In particular, the treatment with the somatostatin analog octreotide (OCT) has been found to inhibit apoptotic cell death and avoid vascular endothelial growth factor (VEGF) overexpression in retinal explants exposed to typical diabetic stressors such as hyperglycemia, oxidative stress (OS) or advanced glycation end-products (Amato et al., 2016). OCT has been shown also to maintain the apoptosis-autophagy equilibrium in high glucose conditions by promoting the restoration of the autophagic flux in bipolar, amacrine, and ganglion cells (Amato et al., 2018a). OCT may also exert antiangiogenic effects, which may be of importance for the treatment of belated stages of DR characterized by aberrant angiogenesis. Indeed, OCT strongly inhibited VEGF-induced cell proliferation, migration and tubulogenesis in human retinal endothelial cells (HRECs) (Palii et al., 2008), prevented hypoxia-induced VEGF upregulation in retinal explants (Mei et al., 2012), and reduced VEGF expression and angiogenesis in a mouse model of oxygen-induced retinopathy (Dal Monte et al., 2009).

Somatostatin or OCT seem to open interesting possibilities for new DR treatments. However, although systemic administration of these factors could be a simple and non-invasive treatment modality, it is affected by important limitations due to the possible occurrence of adverse side effects. For instance, long-term systemic administration of somatostatin or OCT could provoke gastritis, damage of the gastric mucosa, and focal atrophy (Plockinger et al., 1990). In order to avoid systemic side effects, topical administration routes are generally preferred. For instance, drug delivery through eye drops is a non-invasive and non-stressing modality. However, achievement and maintenance of the effective drug concentration in the retinal microenvironment are hindered by external ocular barriers represented by cornea/sclera and the tear film (Agarwal et al., 2016). Moreover, the long-term treatment with eye drops could lead to corneal dryness, inflammation, and damage (Baudouin et al., 2010).

Commonly, diseases of the posterior segment of the eye are treated with drugs administered intravitreally, which ensures the achievement of effective intraocular drug concentrations. However, the long-term maintenance of the effective drug levels requires relatively frequent intravitreal injections, which represent the main limitation of this modality of ocular drug delivery. Indeed, many severe adverse effects may occur after a long period of high frequency intraocular injections, such as retinal detachment, endophthalmitis, cataract, ocular hypertension, and submacular hemorrhages (Krishnan et al., 2009; Adelman et al., 2010).

Biocompatible nanoparticle technology has emerged as a promising drug delivery system allowing specific drug binding/entrapment, enhancing long-term drug release, and reducing time-dependent drug biodegradation (Singh and Lillard, 2009; Lee and Yeo, 2015; Patra et al., 2018). Generally, nanoparticles are designed to be biocompatible, non-antigenic and highly hydrophilic in nature (Li et al., 2012; Naahidi et al., 2013). Different types of nanoparticles have been tested as carriers for the intraocular delivery of drugs inhibiting choroidal or intraretinal neovascularization, thus indicating therapeutic potential for age-related macular degeneration (Park et al., 2009; Liu et al., 2011; Cai and McGinnis, 2016), while nanoparticle-based approaches for the treatment of DR have also been investigated (Fangueiro et al., 2015). Among nanoparticles, magnetic nanoparticles (MNPs) may represent a promising perspective for intraocular drug delivery (Giannaccini et al., 2017b). Indeed, intraocularly injected MNPs have been shown to enter the retina rapidly and persistently localize within the retinal pigment epithelium (RPE) in *Xenopus* and zebrafish without inducing any tissue damage (Giannaccini et al., 2014). Importantly, MNPs have been demonstrated to be effective in loading and delivering molecules such as brain derived neurotrophic factor (BDNF) and nerve growth factor (NGF) to zebrafish eyes with an increase of their effectiveness in preventing oxidative retinal damage (Giannaccini et al., 2018). Here we tested the feasibility of using MNP-bound OCT (MNP-OCT) for treatment of DR. In particular, we assessed the efficacy of MNP-OCT in inhibiting the VEGF-induced proangiogenic responses in HRECs, its effectiveness in protecting *ex vivo* retinal explants from OS-induced apoptosis, and the actual localization of MNP-OCT in mouse retinas *in vivo* after intraocular injection. Preliminary results have been published previously (Amato et al., 2018b).

## Materials and Methods

### Nanoparticle Functionalization

Commercial MNPs were used (FluidMAG-ARA 4115, Chemicell, Berlin, Germany). They are composed by a magnetite core of iron oxide and an organic shell exposing carboxylic groups. Their hydrodynamic diameter is 50 nm (product information sheet). These MNPs are characterized by a polydispersity index of 0.337 ± 0.022, and a negative Z potential (-38.72 ± 2.14 mV) due to their surface functionalization with carboxylic groups (Giannaccini et al., 2018). The nanoparticles were covalently functionalized with OCT (Abcam, Cambridge, United Kingdom) using an MNP/protein ratio of 3.5:1 w/w via EDC chemistry, as previously described (Pinkernelle et al., 2015; Giannaccini et al., 2017a, b). The functionalization procedure was conducted entirely under sterile conditions. Briefly, MNPs were centrifuged (18,000*g*) and resuspended in 4% EDC (Sigma Aldrich, St. Louis, MO, United States) water solution. After 10 min, OCT was added and mixed for 1 h at 4–8°C. Finally, unbound protein was removed by centrifugation (18,000*g*) and supernatant discarding. The amount of MNP-OCT was calculated by subtraction, measuring the protein concentration in the supernatant with the Bradford assay. Conversely, the amount of MNPs was quantified using the thiocyanate assay, according to a protocol published previously (Riggio et al., 2012). Briefly, 3 μl of MNP suspension was resuspended in 50 μl of a solution made of 1 part of 6 M HCl and one part of 65% HNO_3_ (v/v) and incubated at 60°C for 1 h. The sample was water diluted 1:10, an equal volume of 1.5 M KSCN was added, and absorbance recorded at 478 nm. The calibration curve was obtained with a known amount of MNPs (*y* = 0.055x, *R*^2^ = 1), where y is the absorbance at 478 nm and x is the MNP concentration (mg/ml). MNP-OCT were stored in 20% glycerol at −20°C. The final composition of the MNP-OCT water solution was 1 mM OCT + 4,9 mg/mL MNPs + 20% glycerol.

### Transmission Electron Microscopy of MNP-OCT

The distribution, morphology and average size of MNP-OCT were analyzed by transmission electron microscopy (TEM) using a JEM 1010 electron microscope (Jeol, Tokyo, Japan) at 80 kV. TEM samples of MNP-OCT were prepared by placing one drop of a diluted suspension of nanoparticles in ultrapure water on a formvar-carbon-coated copper grid and allowing the solvent to evaporate at room temperature. The average particle size was evaluated by measuring the largest internal dimension of 200 randomly chosen particles using the ruler tool of Adobe Photoshop CS3 (Adobe Systems, Mountain View, CA, United States).

### Cell Culture

*In vitro* studies were performed using HRECs (ACBRI-181, Applied Cell Biology Research Institute, Kirkland, WA, United States). HRECs were cultured in EBM-2 (Lonza, Basel, Switzerland) supplemented with 10% fetal bovine serum (FBS, Sigma Aldrich) and endothelial growth factors (EGM-2MV SingleQuot, Lonza) at 37°C under a humidified 95%:5% (v/v) mixture of air and CO_2_.

### Endothelial Cell Proliferation Assay

HRECs (1 × 10^4^) were starved with EBM-2 containing 0.5% FBS for 18 h to inactivate cell proliferation and successively were treated with or without 40 ng/mL VEGF. OCT bioactivity was tested by adding 1 μM OCT or 1 μM MNP-OCT in the presence or absence of VEGF. We choose the 1 μM concentration because it has been reported to be an effective concentration to counteract VEGF-driven endothelial activation (Palii et al., 2008). In order to test the nanoparticle core toxicity, an equal amount of non-functionalized MNPs (4.9 μg/mL) were added to the culture medium in the presence or absence of VEGF. The dose-response analysis was performed by adding an equal amount of OCT or MNP-OCT following a logarithmic scalar dose correspondent to 1 μM, 0.1 μM, 0.01 μM, or 0.001 μM. After 24 h incubation, the cell viability was quantified spectrophotometrically using the MTT assay (Sigma Aldrich). Absorbance was measured at 595 nm using an iMark microplate reader (Bio-Rad Laboratories, Hercules, CA, United States) for the proliferation rate calculation. Analyses were performed in at least three independent experiments. After statistical analysis, the data from the different experiments were plotted and averaged in the same graph.

### Endothelial Cell Migration Assay

HRECs were allowed to grow to full confluence in 6-well plates pre-coated with 0.1% gelatin and then starved with EBM-2 containing 0.5% FBS for 18 h. The cells were then wounded with pipette tips and washed with PBS. EBM-2 containing 0.5% FBS was added into the wells with or without 80 ng/ml VEGF and treated with OCT, MNPs or MNP-OCT at the concentrations given above. Cell migration toward the wounded area was evaluated after 18 h by using the ImageJ software and an inverted phase contrast microscope (Zeiss, Oberkochen, Germany) equipped with a 10 × objective and a CCD camera. The percentage of the healed area was evaluated in three independent experiments. After statistical analysis, data from the different experiments were plotted and averaged in the same graph.

### Endothelial Cell Tube Formation Assay

The effect of OCT, MNPs, or MNP-OCT on the VEGF-induced HREC tube formation was assessed as previously described (Lulli et al., 2015). Briefly, 100 μl of Matrigel Growth Factor Reduced (Corning, New York, NY, United States) were added to a 96-well plate and allowed to solidify at 37°C for 45 min. 1.5 × 10^4^/well HRECs were starved with EBM-2 containing 0.5% FBS for 18 h and then plated onto the layer of Matrigel in 100 μL of starvation medium with or without 80 ng/mL VEGF and treated with OCT, MPNs or MNP-OCT at the concentrations given above. After 6 h incubation, HREC images were acquired using an inverted microscope equipped with a CCD camera (Zeiss). The area occupied by tubular-like structures was measured using the Angiogenesis Analyzer tool of ImageJ software. Three independent experiments were performed. After statistical analysis, data from the different experiments were plotted and averaged in the same graph.

### Animals

All the procedures were performed in compliance with the ARVO Statement for the Use of Animals in Ophthalmic and Vision Research, the EU Directive (2010/63/EU), and the Italian guidelines for animal care (DL 26/14; Permission number: 132/2019-PR). A total of 30, 4–5 week-old C57BL/6J mice (Envigo, San Pietro al Natisone, Udine, Italy) were used in these studies. In addition, two Balb/c mice of similar age were used for intravitreal injections. The mice were kept in a regulated environment (23 ± 1°C, 50 ± 5% humidity) with a 12 h light/dark cycle (lights on at 8:00 am) with food and water *ad libitum*.

### Retinal Explants

*Ex vivo* cultures were prepared according to published protocols (Amato et al., 2016). Briefly, retinas were dissected, cut into four fragments and transferred onto Millicell-CM culture inserts (Merck Millipore, Burlington, MA, United States) with ganglion cells up. The retinal explants were cultured in 1 mL of serum-free culture medium composed of 50% MEM/HEPES (Sigma Aldrich) containing 6 mM D-glucose, 25% Hank’s buffer salt solution (Sigma Aldrich) 25% PBS, 25 U/mL penicillin, 25 mg/mL streptomycin, 1 μg/mL amphotericin B, and 200 μM L-glutamine. The explants were incubated for 5 days at 37°C under a humidified 95%/5% (v/v) mixture of air and CO_2_. OS treatment consisted in adding H_2_O_2_ to a final concentration of 100 μM. 1 μM OCT, 4.9 μg/mL MNPs, or 1 μM MNP-OCT were added to the culture medium. The medium was changed every other day. The dose-response experiment was performed by adding 1 μM, 0.1 μM, 0.01 μM, or 0.001 μM OCT or equivalent amounts of MNP-OCT to the culture medium. Retinal explants were then incubated for 3 days without changing the medium.

### Immunofluorescence

Retinal explants were fixed in 4% paraformaldehyde in 0.1 M PBS for 2 h and then stored in 25% sucrose in 0.1 M PBS. Ten μm thick sections were cut with a cryostat. Immunofluorescence was performed using a rabbit antibody directed to active caspase-3 (1:500; Sigma Aldrich) and appropriate secondary antibodies conjugated with Alexa Fluor 546 (Molecular Probes, Eugene, OR, United States). Retinal layers were revealed by DAPI counterstain. Digital images were acquired with an epifluorescence microscope (Nikon Eclipse Ni, Nikon Europe, Amsterdam, Netherlands) using a 20x plan apochromat objective. To perform the quantitative analysis, four fragments, each originating from a different retina for experimental condition, were photographed and analyzed. The number of active caspase-3 positive cells was counted in each fragment and normalized for the section length using Adobe Photoshop.

### Quantitative Real-Time PCR

Eight fragments per condition were pooled and total RNA was extracted using an RNA isolation kit (RNeasy Mini Kit; Qiagen, Hilden, Germany) and quantified. First-strand cDNA was generated from 1 μg of total RNA using QuantiTect Reverse Transcription Kit (Qiagen). Quantitative real-time PCR (qPCR) was performed using SsoAdvanced Universal SYBR Green Supermix on a CFX Connect Real-Time PCR Detection System and software CFX manager (Bio-Rad Laboratories). Forward and reverse primers were chosen to hybridize to unique regions of the *caspase-3* gene and of *Rpl13a*, a constitutively expressed gene encoding for ribosomal protein L13A (Mazumder et al., 2003). Primer sequences were as follows:

Caspase-3 forward 5′-GCACTGGAATGTCATCTCGCTC TG-3′;
Caspase-3 reverse 5′-GCCCATGAATGTCTCTCTGAGGT TG-3’;
Rpl13a forward 5′-CACTCTGGAGGAGAAACGGAA GG-3′;
Rpl13a reverse 5′-GCAGGCATGAGGCAAACAG TC-3′.

Samples were compared using the relative threshold cycle (Ct Method). The increase or decrease (x-fold) was determined relative to a control after normalizing to *Rpl13a*. Three pools of fragments per condition, each made of eight retinal fragments originating from different retinas, were analyzed and all reactions were run in duplicate. After statistical analysis, the data from the different experiments were plotted and averaged in the same graph.

### Intravitreal Injections and MNP-OCT Localization in the Retina *in vivo*

Both C57BL/6J and Balb/c mice were employed in the experiments for MNP-OCT localization in the retina after intravitreal injection. In particular, the albino Balb/c mice, in which the RPE is not pigmented, were used to better visualize MNP-OCT localization to the RPE in TEM preparations. The mice were anesthetized with an intraperitoneal injection of Avertin (1.2% tribromoethanol and 2.4% amylene hydrate in distilled water, 0.02 mL/g body weight; Sigma Aldrich). 1 μL of 1 mM MNP-OCT was intravitreally injected using a microsyringe (NanoFil syringe; World Precision Instruments, Sarasota, FL, United States) equipped with a 36 gauge needle. For MNP-OCT localization with Prussian blue staining, C57BL/6J mice were sacrificed 8 h, 24 h or 5 days after the injection (two mice per experimental group). The eyeballs were enucleated, immersion fixed in 4% paraformaldehyde in 0.1 M phosphate buffer for 2 h, and then stored in 25% sucrose in 0.1 M PBS. Subsequently, they were embedded in cryo-gel and 10 μm thick cryostat coronal sections were collected for staining. The RPE was de-pigmented with 0.5% H_2_O_2_ and Prussian blue staining was used to localize the iron oxides composing the nanoparticle core according to the manufacturer’s protocol (Sigma Aldrich). The sections were counterstained with pararosaniline to visualize the retinal layers. For ultrastructural evaluation, two C57BL/6J and two Balb/c mice were sacrificed 24 h after the intravitreal injection. Then the eyeballs were fixed in 2,5% paraformaldehyde and 1% glutaraldehyde and embedded in Epon 812 (Sigma-Aldrich). Semithin sections were cut to evaluate quality and orientation of the tissue. Ultrathin cross-sections of the eye wall were prepared and mounted on grids, stained with UranyLess TEM staining, and examined under a JEM 1010 electron microscope (Jeol) at 80 kV. Images were captured using a CCD digital camera.

### Statistical Analysis

Statistical significance was evaluated with analysis of variance (ANOVA) using the Prism 5.03 software (GraphPad Software, San Diego, CA, United States). The experiments with a single categorical variable (tests of toxicity and OCT bioactivity) were analyzed using one-way ANOVA with Newman–Keuls multiple comparison post-test. The experiments with a double-categorical variable (OCT vs. MNP-OCT dose-response) were analyzed using two-way ANOVA followed by Bonferroni post-test. Differences with *p* < 0.05 were considered significant. The results were expressed as mean ± SEM of the indicated *n* values.

## Results

### TEM Analysis of MNP-OCT

**FIGURE 1:**
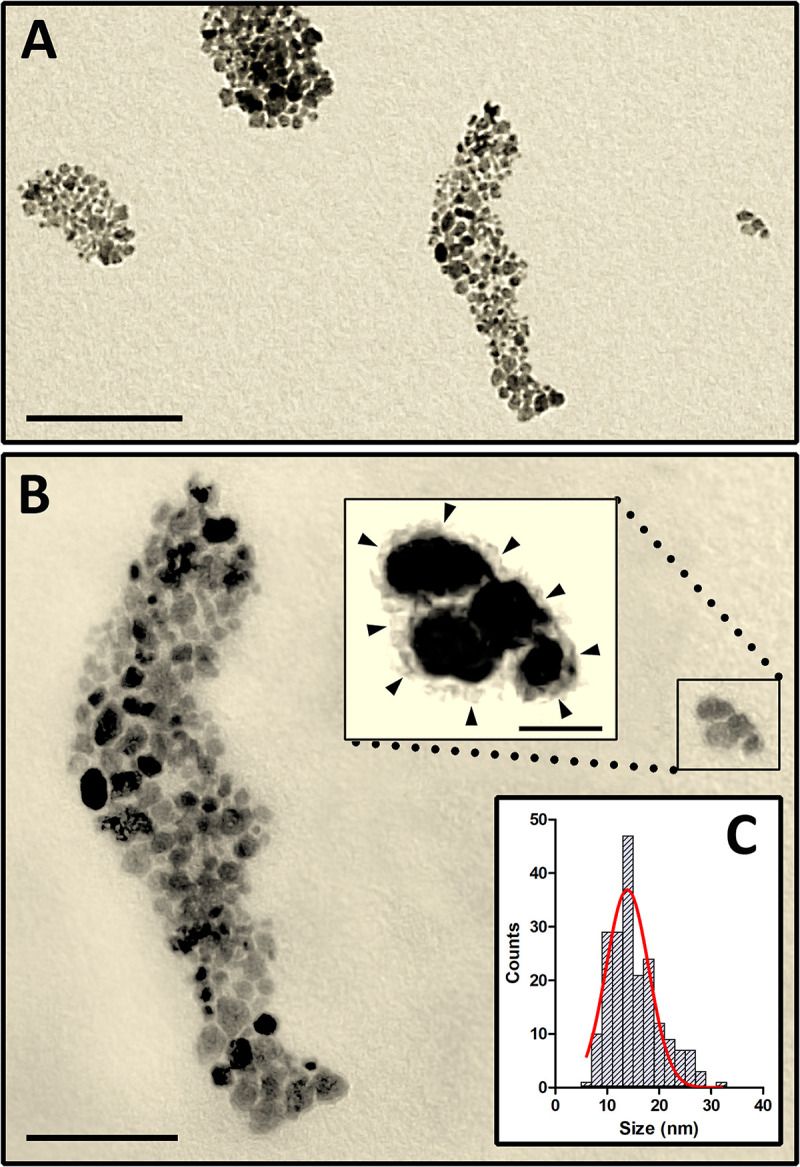
High resolution TEM images showing aggregates of MNP-OCT in a diluted water suspension **(A)**. **(B)** Is a higher power of the right half of **(A)**. The inset is a higher power of the boxed area showing a small group of MNP-OCT. The brightness and contrast of this image were modified with Adobe Photoshop to visualize the organic coating surrounding the magnetite core (arrowheads). **(C)** Shows the histogram and the fitting Gaussian curve used to extract the average particle size. Scale bars, 200 nm in **A**; 100 nm in **B**; 20 nm in the inset.

As observed in TEM, MNP-OCT appeared to form aggregates of various dimensions (Figure 1A). Single MNP-OCT displayed variable sizes and round to oval morphology (Figure 1B). At high magnification and with increased image contrast, the coating composed of an organic shell with the attached OCT around the magnetite core became visible (Figure 1B, inset). The average size of MNP-OCT was calculated counting 200 particles, obtaining the histogram shown in Figure 1C. The histogram could be fitted with a Gaussian distribution (*R*^2^ = 0.84) centered at 13.86 nm (σ = 4.09).

### MNPs Do Not Affect HREC Proliferation, Migration, and Tubulogenesis Induced by VEGF

**FIGURE 2:**
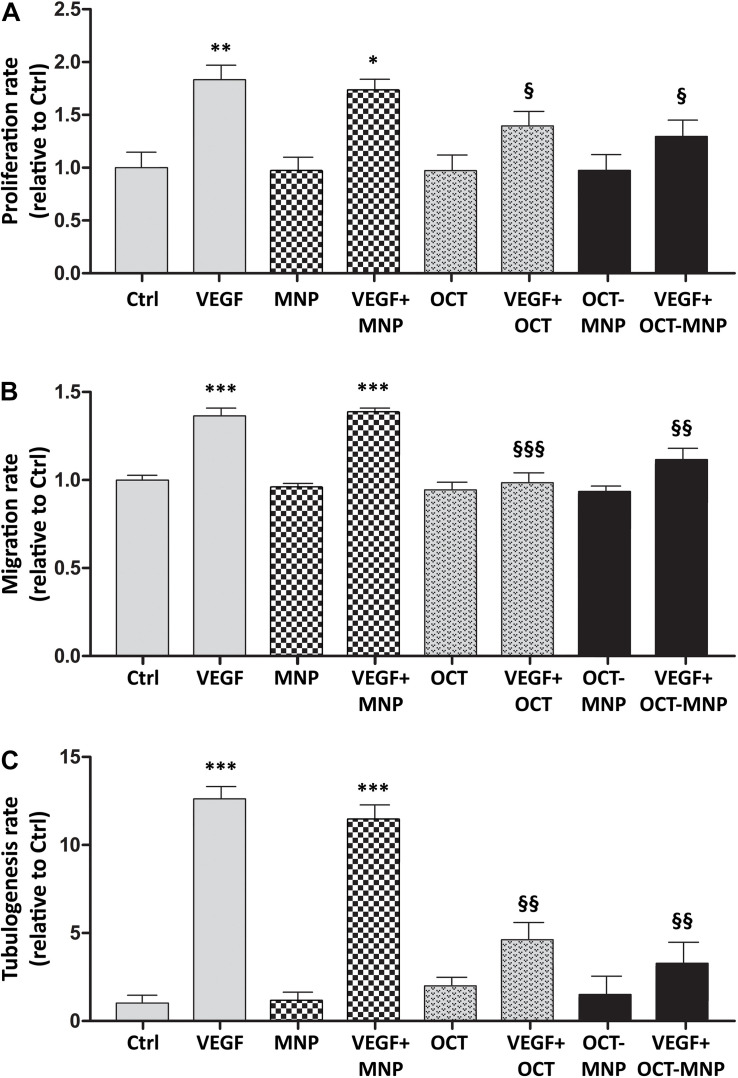
Proliferation **(A)**, migration **(B)**, and tubulogenesis **(C)** of HRECs in control (Ctrl) conditions or in the presence of VEGF (40 ng/mL for proliferation, 80 ng/mL for migration and tubulogenesis) with or without 4,9 μg/mL MNPs, 1 μM OCT or 1 μM MNP-OCT. Values are plotted as means ± SEM. *p < 0.05, **p < 0.01, and ***p < 0.001 vs. Ctrl; ^§^*p* < 0.05, ^§^^§^*p* < 0.01 and ^§^^§^^§^*p* < 0.001 vs. VEGF; *n* = 3.

Untreated HRECs had a basal proliferation rate that was not affected by the presence of MNPs in the incubation medium. As expected, the treatment with VEGF determined an about twofold increment of the cellular proliferation rate, and this response did not change in the presence of MNPs (Figure 2A, checked bars). Similar results were obtained for VEGF-induced migration rate (Figure 2B, checked bars) and tubulogenesis (Figure 2C, checked bars).

### The Binding of OCT to MNPs Does Not Alter OCT Effects on VEGF-Induced HREC Proliferation, Migration, or Tubulogenesis

The inhibitory effect of OCT on VEGF-induced HREC proliferation, migration and tube formation has been well established in previous works (Palii et al., 2008). Our purpose was to test if the covalent binding of OCT to MNP could affect OCT bioactivity. The results showed that 1 μM OCT or MNP-OCT reduced the VEGF-induced increase of HREC proliferation (Figure 2A), migration (Figure 2B), and tube formation (Figure 2C) with the same efficacy.

### VEGF-Induced HREC Proliferation Is Inhibited by MNP-OCT With Greater Efficacy Than OCT

**FIGURE 3:**
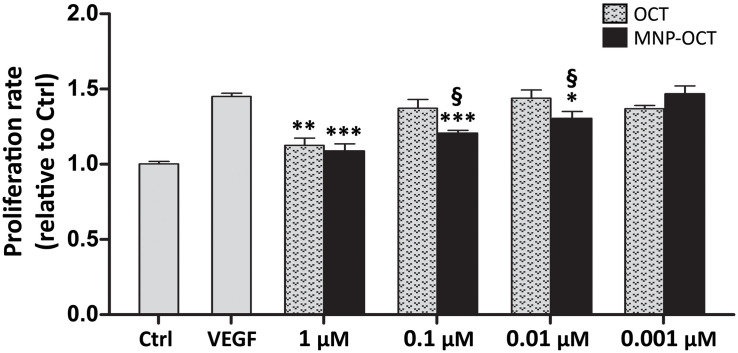
Dose-response of the effects of OCT and of MNP-OCT on HREC proliferation. Data are plotted as mean ± SEM. ^∗^*p* < 0.05, ^∗∗^*p* < 0.01, and ^∗∗∗^*p* < 0.001 vs. VEGF; ^§^*p* < 0.05 vs. OCT; *n* = 4.

We performed a dose-response analysis to evaluate the efficacy of decreasing concentrations of MNP-OCT in inhibiting VEGF-induced HREC proliferation compared to OCT. As shown in Figure 3, we confirmed a significant reduction of VEGF-induced HREC proliferation in the presence of 1 μM OCT; however, at lower concentrations, OCT effects were not significant. In contrast, MNP-OCT were still effective at 0.1 and at 0.01 μM, indicating a much greater efficiency than OCT in inhibiting the VEGF effect on HREC proliferation.

### The Binding of OCT to MNPs Does Not Alter OCT Protective Effects Against OS-Induced Apoptosis in Retinal Explants

**FIGURE 4:**
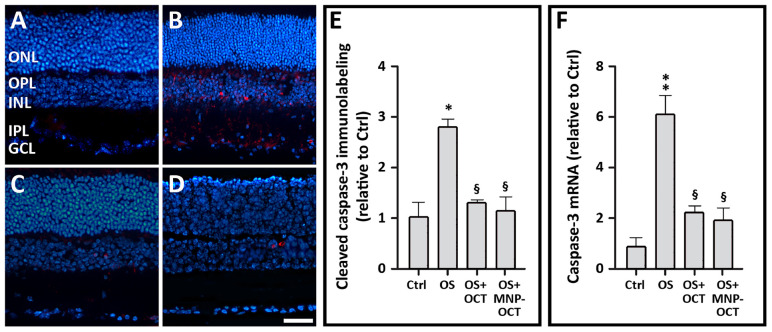
Representative immunofluorescence images of cleaved caspase-3 in sections of retinal explants cultured in control conditions **(A)**, in OS **(B)**, in OS with 1 μM OCT **(C)** or in OS with 1 μM MNP-OCT **(D)**. Retinal layers are visualized with DAPI counterstain. Scale bar, 50 μm. **(E)** Quantitative analysis of the number of cleaved caspase-3 immunopositive cells per unit length of retinal section. **(F)** Quantitative analysis of *caspase-3* mRNA expression as evaluated with qPCR. Values are indicated as mean ± SEM. ^∗^*p* < 0.05 and ^∗∗^*p* < 0.01 vs. the respective Ctrl; ^§^*p* < 0.05 vs. the respective OS; *n* = 3 both in **(E,F)**. GCL, ganglion cell layer; INL, inner nuclear layer; IPL, inner plexiform layer; ONL, outer nuclear layer; OPL, outer plexiform layer.

No differences in retinal layer thickness or general retinal morphology were observed between untreated explants and explants cultured in the presence of MNPs (4.9 μg/mL) alone (not shown). In contrast, active caspase-3 immunostained cells were numerous in the inner nuclear layer (INL) and in the ganglion cell layer (GCL) of retinal explants exposed to OS, and the number of these cells was significantly reduced by treatment with 1 μM OCT or MNP-OCT (Figures 4A–E). In addition, quantification of *caspase 3* mRNA in retinal explants (Figure 4F) confirmed that the treatment with1 μM MNP-OCT had an effect similar to that of 1 μM OCT, demonstrating that OCT bioactivity in OS-exposed retinal explants is not altered by the binding with MNPs.

### OS-Induced Apoptotic Cell Death in Retinal Explants Is Reduced by MNP-OCT With Greater Efficacy Than OCT

**FIGURE 5:**
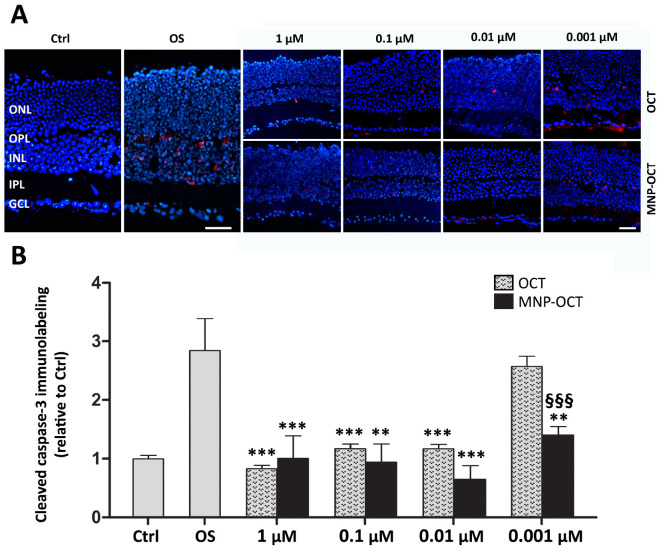
**(A)** Representative immunofluorescence images of cleaved caspase-3 in sections of retinal explants cultured in control conditions (Ctrl), in OS and following OCT or MNP-OCT treatment, as indicated. Retinal layers are visualized with DAPI counterstaining. Scale bar, 50 μm. **(B)** Quantitative analysis of the number of cleaved caspase-3 immunopositive cells per unit length of retinal section. Values are indicated as the mean ± SEM. ^∗∗^*p* < 0.01 and ^∗∗∗^*p* < 0.001 vs. OS; ^§^^§^^§^*p* < 0.001 vs. OCT; *n* = 3. Retinal layer abbreviations as in Figure 4.

As previously reported, 1 μM OCT protects from apoptosis retinal explants exposed to OS (Amato et al., 2016). Similar to the dose-response experiment in HRECs, we performed an experiment in OS-treated retinal explants to assess the efficacy of decreasing concentrations of OCT and of MNP-OCT. Significantly lower numbers of apoptotic cells were observed after treatment with OCT down to a concentration of 0.01 μM, while MNP-OCT were still efficient in inhibiting apoptosis at a concentration of 0.001 μM (Figure 5).

### Retinal Localization of Intravitreally Injected MNP-OCT *in vivo*

**FIGURE 6:**
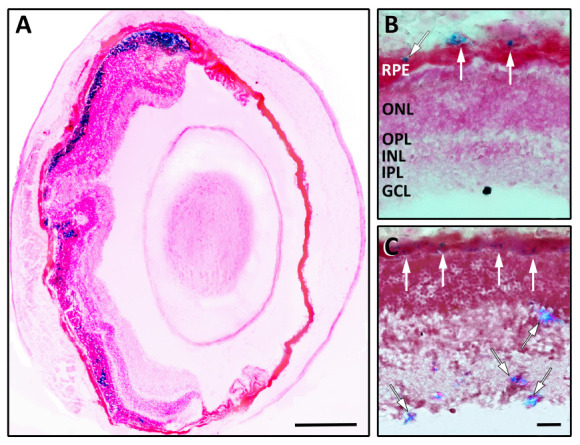
Representative images of C57BL/6J mouse retinal sections after 8 h **(A)**, 24 h **(B)**, and 5 days **(C)** from an intravitreal injection of 1 μM MNP-OCT. The Prussian blue staining identifies the localization of MNP-OCT in the RPE (white arrows in **B**,**C**) and at different levels in the inner retina (black-lined arrows in **C**). Pararosaniline counterstain. Scale bars, 200 μm in **(A)**, 30 μm in **(C)**. Retinal layer abbreviations as in Figure 4.

**FIGURE 7:**
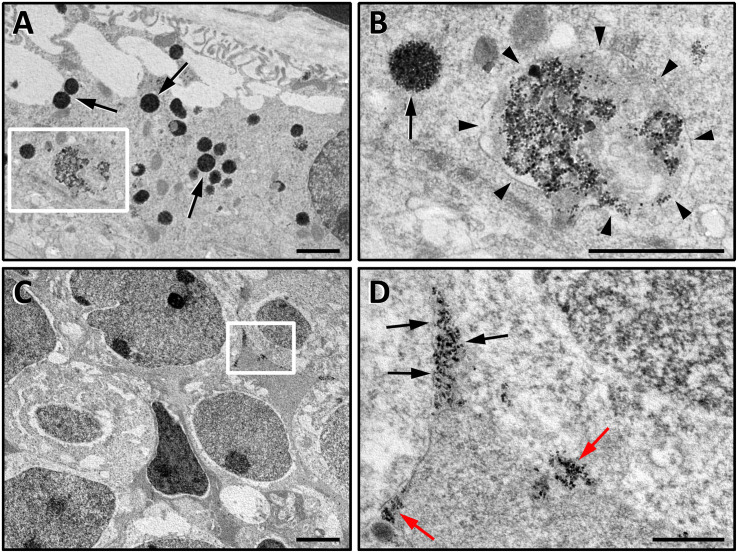
High resolution TEM images of ultrathin retinal sections at the level of the RPE **(A,B)** and of the INL **(C,D)**. Images in **(A,B)** are from a Balb/c mouse retina, in which RPE cells do not contain pigment and therefore MNP-OCT can be easily detected. **(B)** is a higher power of the boxed area in **(A)**. In the RPE, MNP-OCT were found to be internalized in several endocytic vesicles. The scission phase of endocytic process releasing primary endocytic vesicle is highlighted by arrowheads **(B)**, while other MNP-containing vesicles are likely to represent different stages of the following intracellular trafficking (**A,B**, arrows). **(D)** is a higher power of the boxed area in **(C)**. The nanoparticles in the INL were densely packed into narrow intercellular spaces (**D**, black arrows) or distributed in dense spots adjacent to the extracellular side of the plasma membrane of INL cells (**D**, red arrows). Scale bars, 1 μm in (**A**,**B**,**D)**; 2 μm in **(C)**.

The detection of MNPs using Prussian blue histological staining 8 h after the intravitreal injection revealed that MNP-OCT mostly localized to the outer retina in close proximity to the RPE. The density of the staining appeared asymmetrically distributed, probably due to the position of the syringe tip in the vitreous at the moment of the injection (Figure 6A). MNP-OCT were almost exclusively localized to the RPE at 24 h after the injection, and the staining appeared weaker than at 8 h and concentrated in dense spots (Figure 6B). After 5 days from the injection, blue spots revealing the presence of MNP-OCT were also detected within inner retinal layers, and in particular in the INL and GCL (Figure 6C). The ultrastructural analysis confirmed the presence of MNP-OCT in the RPE 24 h after the injection, thereby confirming the observations in the Prussian blue preparations. These data also confirmed uptake of MNP-OCT by RPE cells, since a large amount of nanoparticles was internalized in different types of endocytic vesicles (Figures 7A,B). In addition, MNP-OCT were also detected in the extracellular space among cells of the INL, indicating that at this time some MNP-OCT was still moving across the retina (Figure 7C). In particular, MNP-OCT were observed to form dense accumulation spots probably due to the diffusion of MNP-OCT through narrow intercellular spaces, while several nanoparticles appeared in intimate contact with the plasma membrane of INL cells (Figure 7D). No evidence of MNP-OCT internalization was found in these cells, although MNP-OCT internalization by neurons or glial cells at later times cannot be excluded.

## Discussion

The use of nanoparticles as a drug delivery technology for the treatment of ocular diseases such as DR is receiving increasing attention (Amato et al., 2018b; Bisht et al., 2018; Jiang et al., 2018; Huang and Chau, 2019; Wong and Wong, 2019). The present study investigated the feasibility of using MNPs for the delivery of the somatostatin analog OCT to mammalian retinas. The results show that MNPs are not toxic for retinal cells and that the conjugation of OCT with MNPs not only does not alter, but also significantly improves OCT bioactivity. In addition, intravitreally injected MNP-OCT are localized to the RPE and inner retina up to 5 days post-injection.

### Biocompatibility of MNP-OCT

Several reports have demonstrated that the size and the material of the nanoparticle strongly influence nanoparticle biocompatibility. For instance, an analysis of the toxic responses of rat liver cells has revealed that nanoparticles of different sizes and characterized by different core compositions differentially affect cell viability and function demonstrating that silver nanoparticles are highly toxic whereas aluminum, iron oxide, manganese oxide, and tungsten nanoparticles display little or no toxicity (Hussain et al., 2005). MNPs coated with polyethylenoxide copolymers have been shown to be devoid of adverse effects for the proliferation of epithelial, endothelial, or tumor cells up to a concentration of 5 mg/mL (Hafeli et al., 2009). Regarding the possible use of MNPs in the eye, there are data in experimental animals showing that MNPs are non-toxic to ocular tissues (Raju et al., 2011). Our results show that MNPs do not affect HREC vitality, nor their proliferation, migration or tube formation in response to VEGF. The biocompatibility of MNPs was further confirmed in *ex vivo* mouse retinal explants, where no apoptotic activity attributable to the presence of MNPs was observed. In addition, necrotic phenomena could be excluded since no evidence of structural alterations of retinal layers was recorded. Together, these data demonstrate that MNPs, at the concentration used in our experiments, do not cause toxic effects on either the vascular or the neural components of the retina.

### Bioactivity of MNP-OCT

The functionalization of MNPs with other molecules consists in a series of chemical reactions leading to the binding (either covalent or non-covalent) of the drug to the MNP. Therefore, it is reasonable to imagine that modifications of the drug molecular structure and bioactivity could occur. Indeed, it has been shown that the choice of the functionalization method is crucial for the maintenance of the drug bioactivity (Pinkernelle et al., 2015). For instance, the covalent binding of BDNF or VEGF to MNPs does not affect drug bioactivity, while NGF binding does, as demonstrated in retinas of zebrafish larvae (Giannaccini et al., 2018). We compared the bioactivity of MNP-OCT with that of free OCT, showing that, after binding with MNPs, OCT maintains its antiangiogenic potential inhibiting the VEGF-driven activation of HRECs. Likewise, the antiapoptotic activity of OCT in OS-stressed retinal explants is preserved in MNP-OCT. Hence, our data demonstrate that the covalent binding of OCT to MNPs does not induce any alteration of the drug bioactivity.

### Efficacy of MNP-OCT Compared to That of OCT

Interestingly, the comparison of the efficacy between MNP-OCT and OCT revealed that the conjugation of OCT to MNPs determined a significant improvement of the dose-dependent inhibitory effect of OCT on the HREC response to VEGF and on the OS-induced apoptosis in mouse retinal explants. Indeed, the significant effect of MNP-OCT is maintained at lower doses (100-fold in HRECs and 10-fold in retinal explants) compared to free OCT, demonstrating a strong improvement of the drug bioactivity following binding to MNPs. Of note, the anti-apoptotic effect of MNP-OCT in OS-treated retinal explants was still detected at the lowest dose employed in these experiments, therefore the possibility exists that MNP-OCT may have efficacy even at lower concentrations than 0.001 μM. These observations are consistent with similar results obtained in retinas of OS-treated zebrafish larvae (Giannaccini et al., 2018). The increased efficacy of MNP-OCT is likely to be due to the fact that the conjugation of the drug to MNPs increases its stability, thus improving its activity. Indeed, there are reports in the literature showing that growth factors, such as NGF, glia-derived neurotrophic factor, or fibroblast growth factor-2, significantly increase their stability or undergo slower degradation when they are conjugated to iron oxide MNPs, suggesting that MNP binding prolongs protein half-life, thereby enhancing protein activity (Ziv-Polat et al., 2014; Marcus et al., 2015). Based on these considerations, we expect that MNP-OCT may improve OCT efficacy also in other models of DR, more similar to the diabetic condition, as we have observed recently that 1 μM free OCT prevents cell apoptosis in retinal explants treated not only with OS, but also with high glucose or with advanced glycation end-products (Amato et al., 2016).

### Intraocular Localization of MNP-OCT

Our data show that MNP-OCT preferentially localize to the RPE following intravitreal injection in a mouse eye, similar to previous findings in *Xenopus* embryos and zebrafish larvae obtained with unconjugated MNPs or with MNPs conjugated with neurotrophins (Giannaccini et al., 2014, 2018). In particular, our observations are consistent with a migration of the MNP-OCT from the vitreous chamber toward the retina, and from the inner to the outer retina and into the RPE, in a process that is completed within the first 24 h, although high resolution TEM images documented that small quantities of MNP-OCT were still diffusing through the retina at this time. Different from the findings in *Xenopus* and zebrafish, we observed the presence of spots of Prussian blue staining within inner retinal layers 5 days after injection. This may represent a residue of the MNP diffusion within retinal layers to reach the RPE. However, we did not observe such pattern of Prussian blue staining at 24 h post-injection, when all the labeling appeared to be confined within the RPE. Therefore, we cannot exclude the possibility that, after the first phase of migration through the retina, the MNP-OCT diffuse back from the RPE toward the inner retina. If this is the case, the MNPs could contribute to the maintenance of OCT *in situ* and to its release over a prolonged period of time. In this respect, it has been reported that a single intravitreal injection of a nanoformulation of glial cell derived neurotrophic factor was effective in providing the sustained release of the drug in a model of glaucoma, which resulted in protection from cell death of retinal ganglion cells up to 11 weeks after the injection (Checa-Casalengua et al., 2011).

At the ultrastructural level, MNP-OCT were observed inside cells of the RPE. In particular, the RPE cells internalized nanoparticles in endocytic vesicles such as endosomes and no sign of cell damage was revealed, thus confirming the MNP-OCT safety profile. MNP-OCT within the retina, in particular at the level of the INL, were observed to form clusters in the extracellular space, in accordance with a model proposed previously to explain the movement of MNPs through the retina. According to this model, MNPs diffusing into the retinal cell layers would be forced to move along narrow intercellular spaces, which would cause their clusterization. Then these small MNP aggregates would reach the RPE having a micrometric dimension ideal for promoting engulfment by RPE cells (Kimura et al., 1994; Giannaccini et al., 2017b). In addition, some MNP-OCT were observed in intimate contact with the surface of some cells in the INL. This positioning of the MNP-OCT may suggest the presence of somatostatin receptors, which indeed are known to be expressed by different cells types in the INL of mammalian retinas (Thermos, 2003; Casini et al., 2005; Cervia et al., 2008). This observation is particularly interesting since it indicates that OCT may interact with its receptors when still bound to MNP, therefore without necessarily being released from MNP-OCT.

## Conclusion

The data described here are consistent with observations in *Xenopus* and zebrafish, adding the important notion that also in mammals MNPs can constitute efficient drug carriers for intraocular drug administration. In addition to the lack of any toxic effect, a salient feature of MNP-mediated OCT intraocular administration is that this procedure is likely to induce long-term maintenance of the intraocular drug levels, thereby avoiding the need of repeated intravitreal injections, with the consequent drastic decrease of the risk of pathologic side effects. Further analyses will be necessary to establish the amount of OCT that is released in the retina and to assess the persistence of drug effects in the long period. Nevertheless, the data presented herein indicate that MNP-OCT may represent an efficient OCT formulation for intraocular delivery and a promising candidate for translation to the clinics.

## Data Availability Statement

The datasets generated for this study are available on request to the corresponding author.

## Ethics Statement

The animal study was reviewed and approved by the Italian Ministry of Health (Permission number: 132/2019-PR).

## Author Contributions

ML and GC designed the study. ML performed the *in vitro* experiments. RA and GC performed the *ex vivo* and the *in vivo* experiments. VR prepared the functionalized nanoparticles. MG provided the data of nanoparticle localization in the mouse retina. AP performed the TEM experiments. MD and MC collaborated to data analysis and preparation of manuscript. RA, ML, and GC wrote the manuscript.

## Conflict of Interest

The authors declare that the research was conducted in the absence of any commercial or financial relationships that could be construed as a potential conflict of interest.

## References

[B1] Adelman R. A. Zheng Q. Mayer H. R. (2010). Persistent ocular hypertension following intravitreal bevacizumab and ranibizumab injections. *J. Ocul. Pharmacol. Ther.* 26 105–110. 10.1089/jop.2009.0076 20187807

[B2] Agarwal R. Iezhitsa I. Agarwal P. Abdul Nasir N. A. Razali N. Alyautdin R. (2016). Liposomes in topical ophthalmic drug delivery: an update. *Drug Deliv.* 23 1075–1091. 10.3109/10717544.2014.943336 25116511

[B3] Amato R. Biagioni M. Cammalleri M. Dal Monte M. Casini G. (2016). VEGF as a survival factor in *Ex Vivo* models of early diabetic retinopathy. *Invest. Ophthalmol. Vis. Sci.* 57 3066–3076. 10.1167/iovs.16-19285 27286364

[B4] Amato R. Catalani E. Dal Monte M. Cammalleri M. Di Renzo I. Perrotta C. (2018a). Autophagy-mediated neuroprotection induced by octreotide in an ex vivo model of early diabetic retinopathy. *Pharmacol. Res.* 128 167–178. 10.1016/j.phrs.2017.09.022 28970178

[B5] Amato R. Dal Monte M. Lulli M. Raffa V. Casini G. (2018b). Nanoparticle-mediated delivery of neuroprotective substances for the treatment of diabetic retinopathy. *Curr. Neuropharmacol.* 16 993–1003. 10.2174/1570159X15666170717115654 28714394 PMC6120116

[B6] Baudouin C. Labbe A. Liang H. Pauly A. Brignole-Baudouin F. (2010). Preservatives in eyedrops: the good, the bad and the ugly. *Prog. Retin. Eye Res.* 29 312–334. 10.1016/j.preteyeres.2010.03.001 20302969

[B7] Bisht R. Mandal A. Jaiswal J. K. Rupenthal I. D. (2018). Nanocarrier mediated retinal drug delivery: overcoming ocular barriers to treat posterior eye diseases. *Wiley Interdiscip. Rev. Nanomed. Nanobiotechnol.* 10:20. 10.1002/wnan.1473 28425224

[B8] Cai X. McGinnis J. F. (2016). Nanoceria: a potential therapeutic for Dry AMD. *Adv. Exp. Med. Biol.* 854 111–118. 10.1007/978-3-319-17121-0_16 26427401

[B9] Casini G. Catalani E. Dal Monte M. Bagnoli P. (2005). Functional aspects of the somatostatinergic system in the retina and the potential therapeutic role of somatostatin in retinal disease. *Histol. Histopathol.* 20 615–632. 10.14670/HH-20.615 15736065

[B10] Cervia D. Casini G. Bagnoli P. (2008). Physiology and pathology of somatostatin in the mammalian retina: a current view. *Mol. Cell. Endocrinol.* 286 112–122. 10.1016/j.mce.2007.12.009 18242820

[B11] Cervia D. Catalani E. Casini G. (2019). Neuroprotective peptides in retinal disease. *J. Clin. Med.* 8:1146. 10.3390/jcm8081146 31374938 PMC6722704

[B12] Checa-Casalengua P. Jiang C. Bravo-Osuna I. Tucker B. A. Molina-Martinez I. T. Young M. J. (2011). Retinal ganglion cells survival in a glaucoma model by GDNF/Vit E PLGA microspheres prepared according to a novel microencapsulation procedure. *J. Control. Release* 156 92–100. 10.1016/j.jconrel.2011.06.023 21704662

[B13] Dal Monte M. Ristori C. Cammalleri M. Bagnoli P. (2009). Effects of somatostatin analogues on retinal angiogenesis in a mouse model of oxygen-induced retinopathy: involvement of the somatostatin receptor subtype 2. *Invest. Ophthalmol. Vis. Sci.* 50 3596–3606. 10.1167/iovs.09-3412 19324858

[B14] Fangueiro J. F. Silva A. M. Garcia M. L. Souto E. B. (2015). Current nanotechnology approaches for the treatment and management of diabetic retinopathy. *Eur. J. Pharm. Biopharm.* 95 307–322. 10.1016/j.ejpb.2014.12.023 25536109

[B15] Gabriel R. (2013). Neuropeptides and diabetic retinopathy. *Br. J. Clin. Pharmacol.* 75 1189–1201. 10.1111/bcp.12003 23043302 PMC3635589

[B16] Giannaccini M. Calatayud M. P. Poggetti A. Corbianco S. Novelli M. Paoli M. (2017a). Magnetic nanoparticles for efficient delivery of growth factors: stimulation of peripheral nerve regeneration. *Adv. Healthc. Mater.* 6:1601429. 10.1002/adhm.201601429 28156059

[B17] Giannaccini M. Giannini M. Calatayud M. P. Goya G. F. Cuschieri A. Dente L. (2014). Magnetic nanoparticles as intraocular drug delivery system to target retinal pigmented epithelium (RPE). *Int. J. Mol. Sci.* 15 1590–1605. 10.3390/ijms15011590 24451140 PMC3907888

[B18] Giannaccini M. Pedicini L. De Matienzo G. Chiellini F. Dente L. Raffa V. (2017b). Magnetic nanoparticles: a strategy to target the choroidal layer in the posterior segment of the eye. *Sci. Rep.* 7:43092. 10.1038/srep43092 28256525 PMC5335660

[B19] Giannaccini M. Usai A. Chiellini F. Guadagni V. Andreazzoli M. Ori M. (2018). Neurotrophin-conjugated nanoparticles prevent retina damage induced by oxidative stress. *Cell Mol. Life Sci.* 75 1255–1267. 10.1007/s00018-017-2691-x 29098325 PMC5843686

[B20] Hafeli U. O. Riffle J. S. Harris-Shekhawat L. Carmichael-Baranauskas A. Mark F. Dailey J. P. (2009). Cell uptake and *in vitro* toxicity of magnetic nanoparticles suitable for drug delivery. *Mol. Pharm.* 6 1417–1428. 10.1021/mp900083m 19445482

[B21] Hernandez C. Simo-Servat O. Simo R. (2014). Somatostatin and diabetic retinopathy: current concepts and new therapeutic perspectives. *Endocrine* 46 209–214. 10.1007/s12020-014-0232-z 24627166

[B22] Huang X. Chau Y. (2019). Intravitreal nanoparticles for retinal delivery. *Drug Discov. Today* 24 1510–1523. 10.1016/j.drudis.2019.05.005 31102730

[B23] Hussain S. M. Hess K. L. Gearhart J. M. Geiss K. T. Schlager J. J. (2005). *In vitro* toxicity of nanoparticles in BRL 3A rat liver cells. *Toxicol. In Vitro* 19 975–983. 10.1016/j.tiv.2005.06.034 16125895

[B24] Jiang S. Franco Y. L. Zhou Y. Chen J. (2018). Nanotechnology in retinal drug delivery. *Int. J. Ophthalmol.* 11 1038–1044. 10.18240/ijo.2018.06.23 29977820 PMC6010371

[B25] Kimura H. Ogura Y. Moritera T. Honda Y. Tabata Y. Ikada Y. (1994). *In-Vitro* phagocytosis of polylactide microspheres by retinal-pigment epithelial-cells and intracellular drug-release. *Curr. Eye Res.* 13 353–360. 10.3109/02713689409167299 8055699

[B26] Krishnan R. Goverdhan S. Lochhead J. (2009). Submacular haemorrhage after intravitreal bevacizumab compared with intravitreal ranibizumab in large occult choroidal neovascularization. *Clin. Exp. Ophthalmol.* 37 384–388. 10.1111/j.1442-9071.2009.02043.x 19594565

[B27] Lee J. H. Yeo Y. (2015). Controlled drug release from pharmaceutical nanocarriers. *Chem. Eng. Sci.* 125 75–84. 10.1016/j.ces.2014.08.046 25684779 PMC4322773

[B28] Li X. M. Wang L. Fan Y. B. Feng Q. L. Cui F. Z. (2012). Biocompatibility and toxicity of nanoparticles and nanotubes. *J. Nanomater.* 2012:548389. 10.1155/2012/548389

[B29] Liu H. A. Liu Y. L. Ma Z. Z. Wang J. C. Zhang Q. (2011). A lipid nanoparticle system improves siRNA efficacy in RPE cells and a laser-induced murine CNV model. *Invest. Ophthalmol. Vis. Sci.* 52 4789–4794. 10.1167/iovs.10-5891 21519028

[B30] Lulli M. Cammalleri M. Fornaciari I. Casini G. Dal Monte M. (2015). Acetyl-11-keto-beta-boswellic acid reduces retinal angiogenesis in a mouse model of oxygen-induced retinopathy. *Exp. Eye Res.* 135 67–80. 10.1016/j.exer.2015.04.011 25913458

[B31] Marcus M. Skaat H. Alon N. Margel S. Shefi O. (2015). NGF-conjugated iron oxide nanoparticles promote differentiation and outgrowth of PC12 cells. *Nanoscale* 7 1058–1066. 10.1039/c4nr05193a 25473934

[B32] Mazumder B. Sampath P. Seshadri V. Maitra R. K. DiCorleto P. E. Fox P. L. (2003). Regulated release of L13a from the 60S ribosomal subunit as a mechanism of transcript-specific translational control. *Cell* 115 187–198. 10.1016/s0092-8674(03)00773-6 14567916 PMC13188775

[B33] Mei S. Cammalleri M. Azara D. Casini G. Bagnoli P. Dal Monte M. (2012). Mechanisms underlying somatostatin receptor 2 down-regulation of vascular endothelial growth factor expression in response to hypoxia in mouse retinal explants. *J. Pathol.* 226 519–533. 10.1002/path.3006 21960021

[B34] Naahidi S. Jafari M. Edalat F. Raymond K. Khademhosseini A. Chen P. (2013). Biocompatibility of engineered nanoparticles for drug delivery. *J. Control. Release* 166 182–194. 10.1016/j.jconrel.2012.12.013 23262199

[B35] Palii S. S. Afzal A. Shaw L. C. Pan H. Caballero S. Miller R. C. (2008). Nonpeptide somatostatin receptor agonists specifically target ocular neovascularization via the somatostatin type 2 receptor. *Invest. Ophthalmol. Vis. Sci.* 49 5094–5102. 10.1167/iovs.08-2289 18599562

[B36] Park K. Chen Y. Hu Y. Mayo A. S. Kompella U. B. Longeras R. (2009). Nanoparticle-mediated expression of an angiogenic inhibitor ameliorates ischemia-induced retinal neovascularization and diabetes-induced retinal vascular leakage. *Diabetes* 58 1902–1913. 10.2337/db08-1327 19491211 PMC2712783

[B37] Patra J. K. Das G. Fraceto L. F. Campos E. V. R. Rodriguez-Torres M. D. P. Acosta-Torres L. S. (2018). Nano based drug delivery systems: recent developments and future prospects. *J. Nanobiotechnol.* 16:71. 10.1186/s12951-018-0392-8 PMC6145203 30231877

[B38] Pinkernelle J. Raffa V. Calatayud M. P. Goya G. F. Riggio C. Keilhoff G. (2015). Growth factor choice is critical for successful functionalization of nanoparticles. *Front. Neurosci.* 9:305. 10.3389/fnins.2015.00305 26388717 PMC4557102

[B39] Plockinger U. Dienemann D. Quabbe H. J. (1990). Gastrointestinal side-effects of octreotide during long-term treatment of acromegaly. *J. Clin. Endocrinol. Metab.* 71 1658–1662. 10.1210/jcem-71-6-1658 2229321

[B40] Raju H. B. Hu Y. Vedula A. Dubovy S. R. Goldberg J. L. (2011). Evaluation of magnetic micro- and nanoparticle toxicity to ocular tissues. *PLoS One* 6:26. 10.1371/journal.pone.0017452 21637340 PMC3102660

[B41] Riggio C. Calatayud M. P. Hoskins C. Pinkernelle J. Sanz B. Torres T. E. (2012). Poly-l-lysine-coated magnetic nanoparticles as intracellular actuators for neural guidance. *Int. J. Nanomed.* 7 3155–3166. 10.2147/Ijn.S28460 22811603 PMC3394465

[B42] Simo-Servat O. Hernandez C. Simo R. (2018). Somatostatin and diabetic retinopathy: an evolving story. *Endocrine* 60 1–3. 10.1007/s12020-018-1561-0 29464407

[B43] Singh R. Lillard J. W. Jr. (2009). Nanoparticle-based targeted drug delivery. *Exp. Mol. Pathol.* 86 215–223. 10.1016/j.yexmp.2008.12.004 19186176 PMC3249419

[B44] Szabadfi K. Pinter E. Reglodi D. Gabriel R. (2014). Neuropeptides, trophic factors, and other substances providing morphofunctional and metabolic protection in experimental models of diabetic retinopathy. *Int. Rev. Cell. Mol. Biol.* 311 1–121. 10.1016/B978-0-12-800179-0.00001-5 24952915

[B45] Thermos K. (2003). Functional mapping of somatostatin receptors in the retina: a review. *Vis. Res.* 43 1805–1815. 10.1016/S0042-6989(03)00169-X 12826104

[B46] Wong C. W. Wong T. T. (2019). Posterior segment drug delivery for the treatment of exudative age-related macular degeneration and diabetic macular oedema. *Br. J. Ophthalmol.* 103 1356–1360. 10.1136/bjophthalmol-2018-313462 31040133

[B47] Ziv-Polat O. Shahar A. Levy I. Skaat H. Neuman S. Fregnan F. (2014). The role of neurotrophic factors conjugated to iron oxide nanoparticles in peripheral nerve regeneration: *in vitro* studies. *Biomed. Res. Int.* 2014:267808. 10.1155/2014/267808 25133160 PMC4123480

