# Correction: Association of the somatostatin analog octreotide with magnetic nanoparticles for intraocular delivery: a possible approach for the treatment of diabetic retinopathy

**DOI:** 10.3389/fbioe.2026.1872073

**Published:** 2026-07-03

**Authors:** Rosario Amato, Martina Giannaccini, Massimo Dal Monte, Maurizio Cammalleri, Alessandro Pini, Vittoria Raffa, Matteo Lulli, Giovanni Casini

**Affiliations:** 1 Department of Biology, University of Pisa, Pisa, Italy; 2 Interdepartmental Research Center Nutrafood “Nutraceuticals and Food for Health”, University of Pisa, Pisa, Italy; 3 Department of Experimental and Clinical Medicine, University of Florence, Florence, Italy; 4 Department of Experimental and Clinical Biomedical Sciences “Mario Serio”, University of Florence, Florence, Italy

**Keywords:** mammalian retina, pigment epithelium, endothelial cells, retinal explants, apoptosis, biocompatibility, bioactivity

There were mistakes in Figures 4–6 as published. Preliminary versions of these figures were included erroneously in the final version of the manuscript. The corrected figures appear below.

**FIGURE 4:**
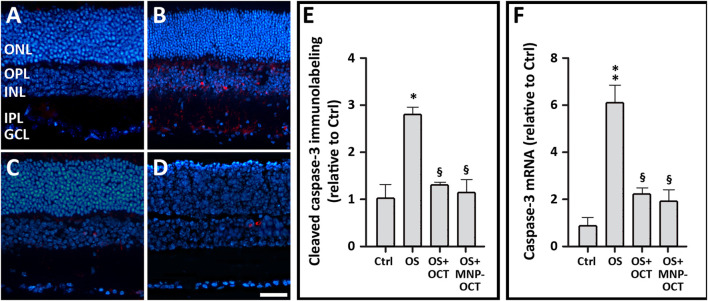
Representative immunofluorescence images of cleaved caspase-3 in sections of retinal explants cultured in control conditions **(A)** in OS **(B)** in OS with 1 µM OCT **(C)** or in OS with 1 µM MNP-OCT **(D)** Retinal layers are visualized with DAPI counterstain. Scale bar, 50 µm. **(E)** Quantitative analysis of the number of cleaved *caspase-3* immunopositive cells per unit length of retinal section. **(F)** Quantitative analysis of caspase-3 mRNA expression as evaluated with qPCR. Values are indicated as mean ± SEM. *p < 0.05 and **p < 0.01 vs. the respective Ctrl; ^§^ *p* < 0.05 vs. the respective OS; *n* = 3 both in **(E,F)**. GCL, ganglion cell layer; INL, inner nuclear layer; IPL, inner plexiform layer; ONL, outer nuclear layer; OPL, outer plexiform layer.

**FIGURE 5:**
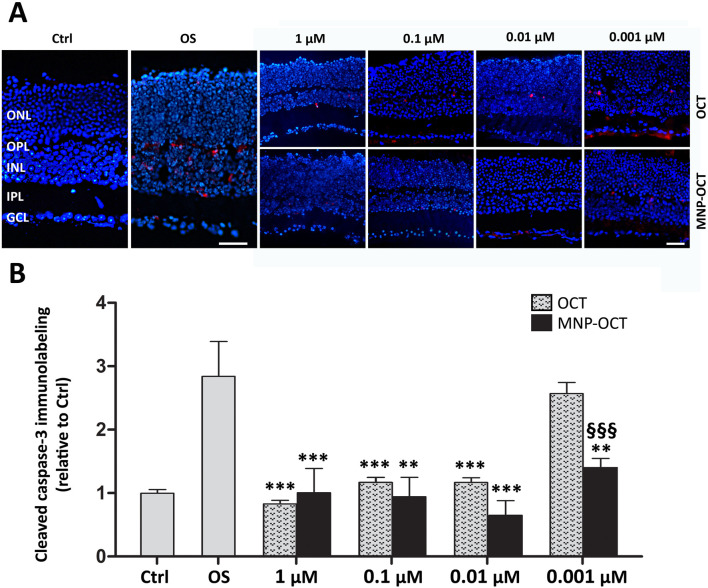
**(A)** Representative immunofluorescence images of cleaved caspase-3 in sections of retinal explants cultured in control conditions (Ctrl), in OS and following OCT or MNP-OCT treatment, as indicated. Retinal layers are visualized with DAPI counterstaining. Scale bar, 50 µm. **(B)** Quantitative analysis of the number of cleaved caspase-3 immunopositive cells per unit length of retinal section. Values are indicated as the mean ± SEM. **p < 0.01 and ***p < 0.001 vs. OS; ^§§§^ *p* < 0.001 vs. OCT; n = 3. Retinal layer abbreviations as in Figure 4.

**FIGURE 6:**
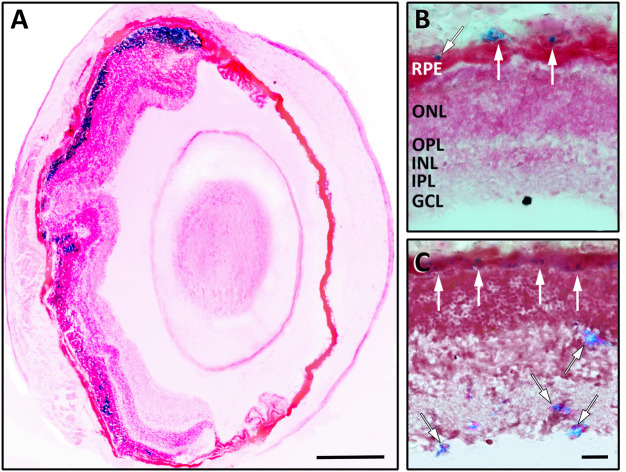
Representative images of C57BL/6J mouse retinal sections after 8 h **(A)** 24 h **(B)** and 5 days **(C)** from an intravitreal injection of 1 µM MNP-OCT. The Prussian blue staining identifies the localization of MNP-OCT in the RPE [white arrows in **(B,C)**] and at different levels in the inner retina (black-lined arrows in **C**). Pararosaniline counterstain. Scale bars, 200 µm in **(A)** 30 µm in **(C)**. Retinal layer abbreviations as in Figure 4.

The original version of this article has been updated.

